# An Outline of the Embryology of the Eye—Prize Essay

**Published:** 1895-04

**Authors:** 


An Outline of the Embryology of the Eye—The Cartwright Prize Essay for 1893. By Mark A. Halden; A. M., M. D., Assistant Surgeon to the New York Opthalmic and Aural Institute; Clinical Assistant Vanderbilt Clinic. New York: G. P. Putnam's Sons

The above is one of the best books we have seen in sometime. Dr. Halden has clearly and elaborately made this subject a pleasure to study under his guidance. The illustrations are from the author's own drawings and are beautiful. The book being printed by the Knickerbocker Press, is guarantee enough of the beauty of the mechanical part. G. B.

